# Four-year visual outcomes and optical quality of SMILE and implantable collamer lens V4c (EVO-ICL) implantation for high myopia: a retrospective study

**DOI:** 10.1186/s12886-023-03050-9

**Published:** 2023-07-31

**Authors:** Wuqiang Luo, Aruma Aruma, Meiyan Li, Jing Wang, Jing Xie, Xin Xiao, Yang Shen, Lingling Niu, Xiaoying Wang, Xingtao Zhou

**Affiliations:** 1 Visual Science and Optometry Center, the People’s Hospital of Guangxi Zhuang Autonomous Region, Nanning, 530021 Guangxi Zhuang Autonomous Region China; 2 Department of Ophthalmology, NHC Key Laboratory of Myopia, Shanghai Research Center of Ophthalmology and Optometry, Eye and ENT Hospital of Fudan University, 200433 Shanghai, China; 3 Aier Eye Hospital Group, Aier Eye Hospital of Wuhan University, 430063 Wuhan, Hubei Province China; 4 Department of Ophthalmology, Shenzhen Key Laboratory of ENT, Institute of ENT Shenzhen, Longgang ENT hospital, 518172 Shenzhen, China; 5 Department of Ophthalmology and Optometry, Myopia Key Laboratory of the Health Ministry, Eye and ENT Hospital of Fudan University, No.83 Fenyang Road, 200031 Shanghai, People’s Republic of China

**Keywords:** High myopia, Small incision lenticule extraction, Implantable collamer lens V4c, Visual quality

## Abstract

**Background:**

To compare the 4-year visual outcomes of implantable collamer lens V4c (EVO-ICL) implantation and small incision lenticule extraction (SMILE) for high myopia and astigmatism.

**Methods:**

This retrospective case study included 64 eyes of 40 patients. These patients with preoperative manifest refraction spherical equivalent (SE) between − 6.00 and − 10.00 diopters (D) were screened from the database of SMILE and EVO-ICL implantation procedures in 2015. The ICL group [32 eyes of 19 patients (mean age, 29.6 ± 6.3 years); mean SE, -8.71 ± 1.06 D] and SMILE group [32 eyes of 21 patients (mean age, 27.7 ± 5.6 years); mean SE, -8.35 ± 0.65D] were compared. All patients were then prospectively examined at a four-year follow-up for routine postoperative examinations, higher-order ocular aberrations, retinal image quality and a questionnaire.

**Results:**

The safety indexes were 1.15 ± 0.14 and 1.22 ± 0.21 (P = 0.36) for the SMILE and ICL groups, respectively. No eyes lost two or more lines of CDVA in either group. The efficacy indexes were 0.97 ± 0.16 and 0.96 ± 0.19 (P = 0.87), respectively. Twenty-three eyes (72%) in ICL and 26 eyes (81%) in SMILE groups were within ± 0.5 D of the attempted SE (P < 0.01). ICL-treated eyes had significantly less spherical aberration and coma (P < 0.01 and < 0.05, respectively) postoperatively. Halos were the prevalent visual disturbance in both groups.

**Conclusion:**

SMILE and EVO-ICL implantation provided safe and effective correction of high myopia. SMILE showed slightly better long-term predictability. Mild postoperative visual disturbances were observed after ICL and SMILE at 4-year follow-up.

**Supplementary Information:**

The online version contains supplementary material available at 10.1186/s12886-023-03050-9.

## Introduction

In the field of refractive surgery, small incision lenticule extraction (SMILE) has gradually become widely accepted for correcting myopia. Instead of a corneal flap, a 2-mm surgical incision maintains the corneal structure stability, reduces the postoperative corneal denervation degree, and avoids flap-related complications [1]. Its safety, efficacy, and predictability have been reported [2–5]. However, corneal refractive surgery, as a “subtraction” operation in which a corneal lenticule is extracted, remains limited by corneal thickness. The implantable collamer lens (ICL™; STAAR Surgical, Nidau, Switzerland) implantation, is an “addition” operation in which an intraocular lens is added. It has a wider range of refractive correction (up to -20.00 D of myopia). Its safety, efficacy, predictability, and stability have been reported [6, 7]. A newly improved and widely accepted ICL with a 360-µm central hole in the central optical zone (EVO-ICL) eliminates the need for preoperative iridotomy and reduces the risk of anterior subcapsular cataract formation [8, 9]. However, visual disturbances, such as halos and glare, were reported early postoperatively after both conventional ICL and EVO-ICL implantation [10–12].

More studies focused on investigating the differences in the visual outcomes of these two procedures. Qin et al. reported that based on the Optical Quality Analysis System (OQAS) II values, the postoperative visual quality of EVO-ICL implantation was slightly better than that of SMILE [13]. EVO-ICL reportedly caused lower higher-order aberrations (HOAs) induction than SMILE [12, 14]. Regarding subjective quality of vision, each technique reportedly resulted in a specific spectrum of visual symptoms at the early postoperative stage in high myopia correction [12, 14].

Li et al. conducted a meta-analysis on the visual quality of ICL and SMILE procedures, with the majority of comparative studies focusing on a follow-up period of 1 year [15]. In this study, we evaluated the long-term (up to 4 years) refractive outcomes and optical quality, objectively and subjectively, after SMILE and EVO-ICL implantation for high myopia.

## Methods

### Patients

This retrospective study evaluated the long-term follow-up findings after EVO-ICL implantation and SMILE. This study was approved by the ethics committee of the Eye and ENT Hospital at Fudan University and was conducted according to the principles of the Declaration of Helsinki. All patients provided written informed consent to use their data for analysis and publication.

Our institution’s database of EVO-ICL implantation and SMILE during 2015 from the Refractive Surgery Center of the Department of Ophthalmology, EENT Hospital of Fudan University (Shanghai, China), was screened. Inclusion criteria were sphere refraction diopters (D) between − 6.00 D and − 9.00 D (including − 6.00 D), astigmatism of up to -3.50 D, preoperative corrected distance visual acuity (CDVA) of 16/20 or better, and age between 19 and 41 years at the time of surgery. Patients with a history of ocular surgery, inflammation or trauma, a history of certain ocular diseases (suspicion of keratectasia, severe dry eye, cornea or lens opacity, glaucoma, retinal detachment or macular degeneration and so on), and certain systemic diseases were excluded.

The experienced surgeons (XZ and WX) classified the patients to undergo either EVO-ICL implantation (ICL group) or SMILE (SMILE group) after evaluating the patients’ eye condition and their preferences. The surgeon chose EVO-ICL implantation over SMILE, if the patient was not a suitable candidate for corneal refractive surgeries owing to relatively thin corneas, abnormal posterior corneal elevation, or other irregularities in the corneal tomography. In the ICL group, eyes with astigmatism of -1.25 D or greater, toric lenses were implanted. For eyes with astigmatism of -0.50 to -1.00 D, spectacle lenses were tried. If patients were satisfied with their distance visual acuity without astigmatism correction, non-toric lenses were implanted; otherwise, toric lenses were implanted.

### Main refractive and biometric measures

Routine measurements before and after the surgery included: (1) Manifest SE, uncorrected distance visual acuity (UDVA), and CDVA measured with a standard logarithmic chart; (2) Slit-lamp biomicroscopic and fundoscopic examinations; (3) IOP by a non-contact tonometer (Tonemeterx-10; Canon, Tokyo, Japan); (4) Anterior chamber depth (ACD), CCT and vault measurement using a Pentacam camera system (Oculus, Germany); (5) Axial length (AL) measurements and horizontal corneal diameter (white-to-white, WTW) using an IOL-Master (Carl Zeiss, Germany); (6) Endothelial cell density (ECD; SP-2000P, Topcon Corporation, Japan).

Follow-up measurements were scheduled at 4 years postoperatively including ocular higher-order aberrations, retinal image quality and intraocular scattering. Aberrational data measured by the WASCA Analyzer (Carl Zeiss, Meditec, Jena, Germany) were analyzed at 5 mm pupil size using Zernike polynomials. The retinal image quality and intraocular scattering surgery were measured with the OQAS II (Visiometrics, Terrassa, Spain), which has good repeatability and reliability [16].

### Subjective visual quality

The incidence and severity of night vision disturbances were evaluated using a questionnaire provieded in supplementary Table 1 which was adminstered by Zhao et al. for measuring the visual quality of patients undergoing SMILE [17]. Recipients were asked to evaluate their current night vision disturbances (glare, halos, starburst and visual distortion) using a 4-level scale of none, mild, moderate, or severe. Their overall satisfaction with the procedure and whether they would recommend EVO-ICL implantation or SMILE to others were also collected.

### Surgical techniques

The EVO-ICL power calculation (STAAR Surgical, Nidau, Switzerland) was performed according to the manufacturer’s instructions using a modified vertex formula based on the preoperative refractive parameters. The size of the implanted EVO-ICL was determined by the value of WTW and ACD. Optical zone diameter was 5.8 mm for 21 eyes with SE between − 6.25 D and − 9.25 D and 5.3 mm for 11 eyes with SE between − 9.5 D and − 10.0 D in the ICL group. The technical procedure was conducted as previously described [18]. All EVO-ICL implantation procedures were performed by two experienced surgeons (XZ and XW).

All patients had bilateral SMILE performed by the same experienced surgeon (XZ) for the correction of myopia or myopic astigmatism using the VisuMax femtosecond laser system (Carl Zeiss Meditec AG, Jena, Germany) with a 500 kHz repetition rate. All eyes followed the settings as pulse energy of 130 nJ, corneal cap thickness of 110–120 μm, and optical zone diameter of 6.0-6.7 mm. The surgical procedure of SMILE has been previously described by Li et al.[19].

### Statistical analysis

Statistical analysis was performed using SPSS (version 22.0, IBM Corp. Al Monk, NY, USA). Normality was tested using the Kolmogorov–Smirnov test. Preoperative and postoperative results were compared using the Wilcoxon signed-rank test. The frequency counts and percentages of participants as categorical data were compared by Chi-square test. The between-group differences of ocular HOAs were estimate by the generalized estimating equation (GEE) model, in order to model the correlation of responses from the same patients. Differences were considered statistically significant at P < 0.05.

## Results

The ICL group included 32 eyes of 19 patients (2 men and 17 women, mean age, 29.6 ± 6.3 years). There were seven eyes with preoperative cylindrical diopters ranging from − 0.75 D to -3.25 D implanted with toric-ICL lenses and 25 eyes with non-toric lenses. The SMILE group included 32 eyes of 21 patients (3 men and 18 women, mean age 27.7 ± 5.6 years). The preoperative demographics are summarized in Table 1.

**Table 1:** Patient demographics and preoperative refractive parameters

Characteristics	EVO-ICL(N = 32)		SMILE (N = 32)		P Value
	MEAN ± SD	RANGE	MEAN ± SD	RANGE	
Age (years)	29.6 ± 6.3	19 to 41	27.7 ± 5.6	19 to 41	0.21
Gender (Female:Male)	17:2	-	18:3	-	-
Manifest spherical equivalent (D)	-8.71 ± 1.06	-10.00 to -6.25	-8.35 ± 0.65	-9.63 to -6.88	0.10
Manifest Sphere (D)	-8.04 ± 0.93	-9.00 to -6.00	-7.84 ± 0.64	-9.00 to -6.00	0.31
Manifest cylinder (D)	-1.32 ± 1.04	0 to -3.50	-1.00 ± 0.45	-0.25 to -2.0	0.12
CCT (µm)	498.2 ± 23.2	462.0 to 558.0	546.1 ± 23.3	486.0 to 600.0	< 0.001
NCT (mmHg)	14.20 ± 2.84	8.30 to 20.10	16.16 ± 2.91	9.90 to 20.50	0.08
AL (mm)	26.72 ± 0.70	25.05 to 27.81	26.49 ± 1.10	24.14 to 28.83	0.25

* EVO-ICL = implantable collamer lens with a central hole, SMILE = femtosecond laser small incision lenticule extraction, SD = standard deviation, D = diopters, CCT = mean central corneal thickness, NCT = non-contact tonometer, AL = axial length, Manifest spherical equivalent, manifest sphere, manifest cylinder using non-parametric test and t-test for age, NCT and AL

* The generalized estimating equation (GEE) model was used to estimate between-group differences of change in aberrations from baseline to one year

There were no significant differences between the groups in terms of age (P = 0.21), manifest spherical equivalent (SE) (P = 0.10), and manifest cylinder (P = 0.12) preoperatively. The mean central corneal thickness of the SMILE group was significantly higher than that of the ICL group (P < 0.001).

### Safety

**Fig. 1:**
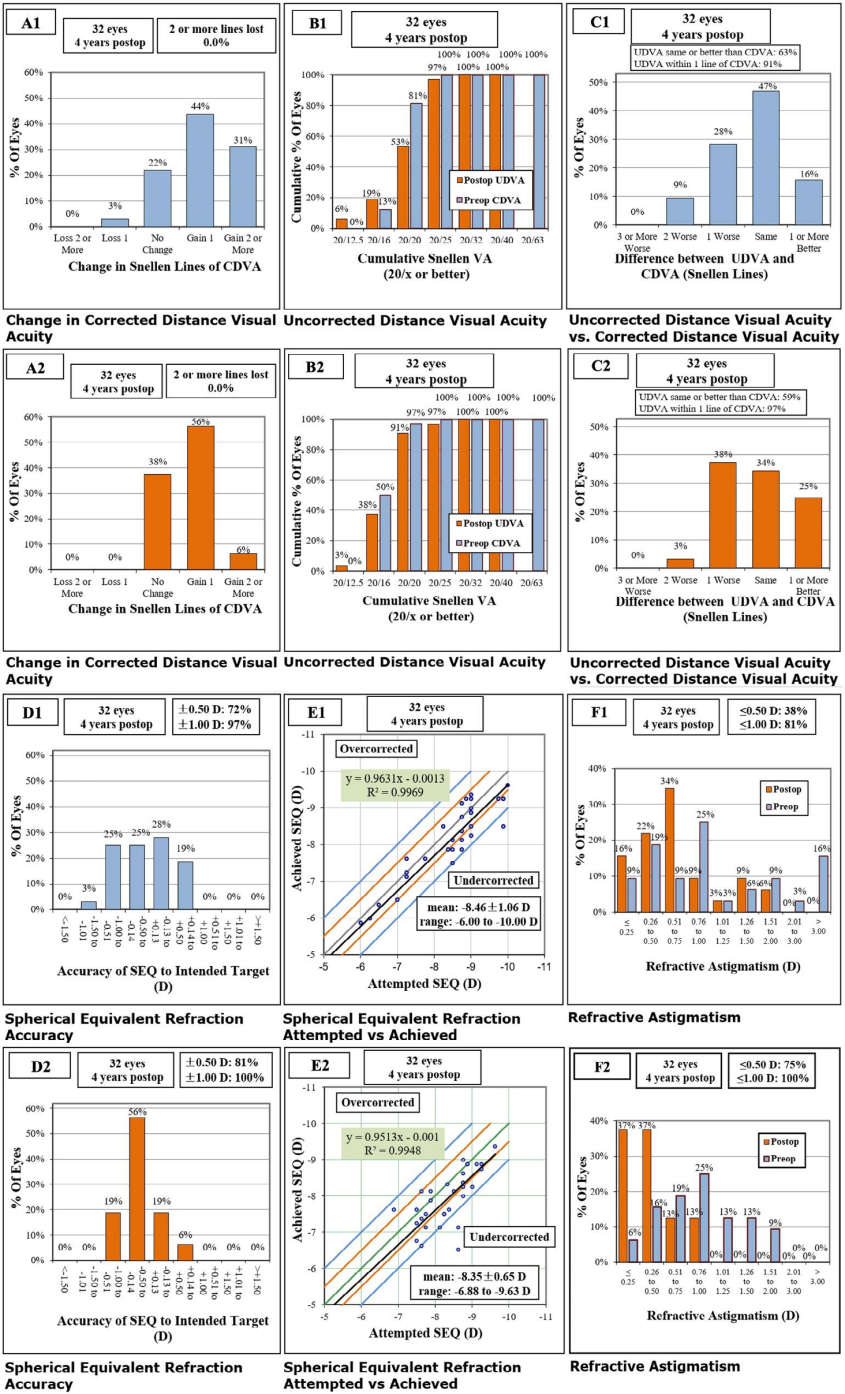
Refractive outcomes 4 years after implantable collamer lens with a central hole (EVO-ICL) implantation and femtosecond laser small incision lenticule extraction (SMILE). (**A1** and **A2**) Change in preoperative corrected distance visual acuity (CDVA) for EVO-ICL implantation and SMILE, respectively; (**B1** and **B2**) Cumulative uncorrected distance visual acuity (UDVA) after EVO-ICL implantation and SMILE, respectively; (**C1** and **C2**) postoperative UDVA versus CDVA for EVO-ICL implantation and SMILE, respectively; (**D1** and **D2**) spherical equivalent (SE) refraction after EVO-ICL implantation and SMILE, respectively; (**E1** and **E2**) Attempted versus achieved SE refraction after EVO-ICL implantation and SMILE, respectively; (**F1** and **F2**) Refractive astigmatism after EVO-ICL implantation and SMILE, respectively

All surgeries were uneventful. No cataract formation, glaucoma, or any other vision-threatening complications were observed during the follow-up period. No eyes lost two or more lines of CDVA in either group (Fig. 1A). One eye (3%) in the ICL group lost one line, whereas no eyes in the SMILE group lost any CDVA. The safety indexes (postoperative CDVA / preoperative CDVA) were 1.15 ± 0.14 and 1.22 ± 0.21 in the SMILE and ICL groups, respectively (P = 0.36).

In the ICL group, the IOPs were 14.2 ± 2.8 mmHg at baseline and 13.9 ± 1.5 mmHg at 4 years postoperatively (P = 0.46). The axial lengths were 26.72 ± 0.70 mm preoperatively and 26.70 ± 1.10 mm at 4 years postoperatively (P = 0.87). The endothelial cell density dropped significantly, from 2822.5 ± 349.4 cells/mm^2^ preoperatively to 2640.5 ± 259.1 cells/mm^2^ 4 years postoperatively (P = 0.01). The mean percentage of endothelial cell loss was 5.6% ± 10.7% (-15.3 to 26.6%). No eyes had an endothelial cell loss that decreased to less than 2000 cell/mm² or had a significant loss over 30%.

### Efficacy

The efficacy of the two procedures is shown in Fig. 1B. Postoperatively, 59% (19 eyes) of SMILE eyes and 63% (20 eyes) of ICL-implanted eyes had UDVA no worse than pre-CDVA (Fig. 1C). The efficacy indexes (postoperative UDVA/preoperative CDVA) were comparable between the SMILE (0.97 ± 0.16) and ICL (0.96 ± 0.19) groups (P = 0.87).

### Predictability

The postoperative SE was − 0.27 ± 0.34 D (range, -0.75 to + 0.50 D) and − 0.55 ± 0.35 D (range, -1.38 to + 0.38 D) in the SMILE and ICL groups, respectively (P = 0.02) (Fig. 1D). Figure 1E shows a scatterplot of the attempted and achieved SE corrections. All eyes (100%) in the SMILE group and 97% eyes in ICL group were within ± 1.00 D of the attempted SE. 81% eyes in the SMILE group and 72% eyes in the ICL group were within ± 0.50 D of the attempted SE.

The postoperative mean residual cylinder was − 0.43 ± 0.35 D and − 0.78 ± 0.44 D in the SMILE and ICL groups, respectively (p < 0.001). Of seven eyes with toric-ICL, five had postoperative astigmatism of ≤-0.5 D and all eyes had postoperative astigmatism of ≤-1.0 D. Meanwhile, among eyes with non-TICL, astigmatism was ≤-0.5 D in 28% (7 eyes) eyes and ≤-1.0 D in 80% (20 eyes) eyes, postoperatively. In the SMILE group, postoperative astigmatism was ≤-0.5 D in 75% (24 eyes) of the eyes, and all eyes achieved postoperative astigmatism of ≤-1.0 D (Fig. 1F).

### Higher order aberrations

Table 2 shows an overview of the postoperative HOAs. SMILE-treated eyes showed significantly higher amounts of spherical aberration and coma than ICL-treated eyes (both P < 0.001). Vertical coma and horizontal coma were significantly less after ICL implantation than after SMILE (P = 0.002, P = 0.01, respectively).

**Table 2:** Postoperative in higher-order aberrations in eyes undergoing EVO-ICL implantation and SMILE

Parameters	Postoperative Aberrations of EVO-ICL	Postoperative aberrations of SMILE	P Value
	MEAN ± SD	MEAN ± SD	
Total HOAs (µm)	0.23 ± 0.12	0.20 ± 0.08	0.24
	range, 0.05 to 0.44	range, 0.08 to 0.41	
Coma (µm)	1.17 ± 0.82	2.88 ± 2.14	< 0.001
	range, 0.25 to 3.37	range, -3.70 to 5.94	
Vertical coma (µm)	0.04 ± 0.13	-0.26 ± 0.48	0.002
	range, -0.18 to 0.39	range, -1.66 to 0.79	
Horizontal coma (µm)	0.09 ± 0.26	0.39 ± 0.57	0.01
	range, -0.12 to 1.10	range, -0.29 to 1.69	
Vertical trefoil (µm)	0.01 ± 0.23	-0.04 ± 0.21	0.45
	range, -0.68 to 0.47	range, -0.54 to 0.46	
Oblique trefoil (µm)	0.01 ± 0.28	0.01 ± 0.19	0.93
	range, -0.88 to 0.93	range, -0.32 to 0.63	
Spherical aberration (µm)	-1.43 ± 1.09	-3.87 ± 2.43	< 0.001
	range, -4.05 to 0.57	range, -7.44 to 3.87	

*EVO-ICL = implantable collamer lens with a central hole, SMILE = femtosecond laser small incision lenticule extraction, HOAs = higher-order aberrations

*Postoperative results were compared using the Wilcoxon signed-rank test

### Optical quality

At 4 year postoperatively, the MTF_cutoff_ in the ICL group was 30.46 ± 10.19 cpd (range, 10.50 to 51.01 cpd), which was comparable with it in the SMILE group (27.95 ± 9.1 cpd; range, 13.99 to 47.50 cpd; P = 0.30). OSI had no statistical difference between the SMILE (1.3 ± 0.5; range, 0.5 to 2.5) and ICL (1.3 ± 0.7; range, 0.3 to 2.8, P = 0.95). In SMILE group, the mean Strehl2D ratio was 0.16 ± 0.04 (range, 0.11 to 0.23), and in ICL it was 0.18 ± 0.06 (range, 0.08 to 0.32; P = 0.06).

### Questionnaire

**Fig. 2:**
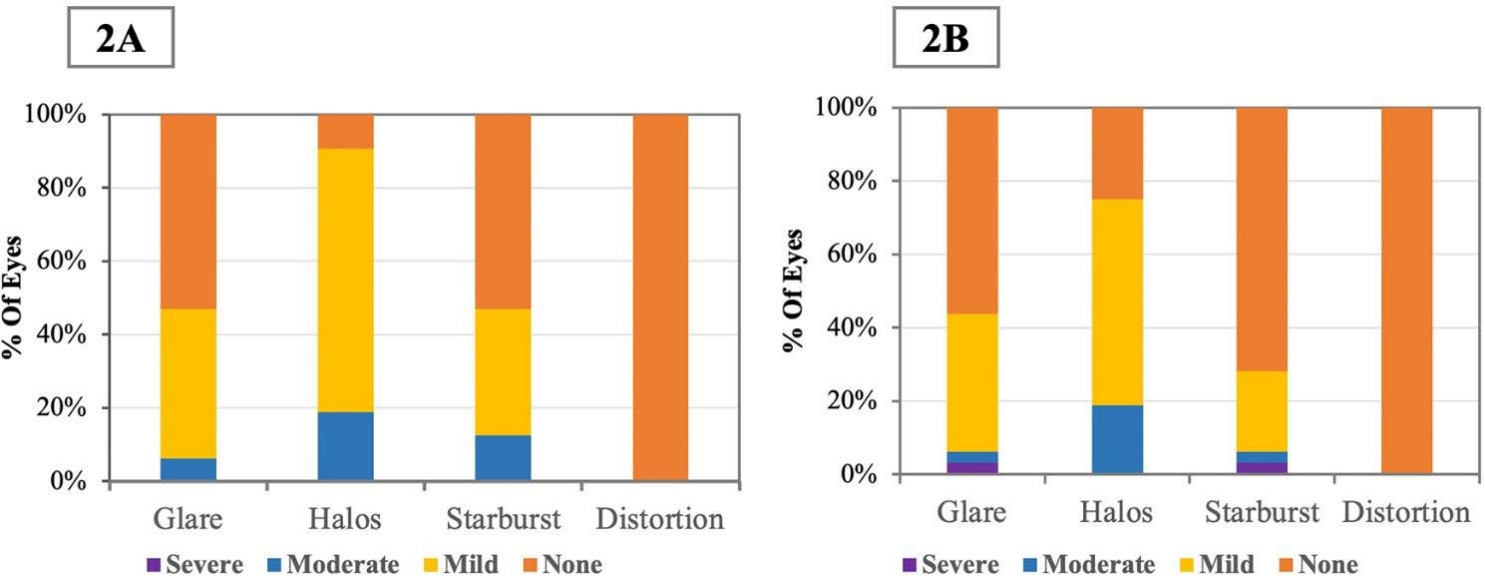
The incidence and severity of night vision disturbances after implantable collamer lens with a central hole (EVO-ICL) implantation (2 **A**) and femtosecond laser small incision lenticule extraction (SMILE; 2**B**)

Of 32 total eyes in the ICL group, 15 (47%) experienced glare, 29 (91%) experienced halos and 15 (47%) experienced starbursts (Fig. 2A). And of all subjects in the SMILE group, 14 (44%) experienced glare, 24 (75%) experienced halos and 9 (28%) experienced starbursts(Fig. 2B). Of all questionnaire responses from ICL-treated eyes, 1 (3%) eye reported more than a moderate degree of halos. Of all questionnaire responses in the SMILE group, 1 (3%) eye, respectively, reported more than a moderate degree of glare and starburst.

Regarding patient satisfaction, 96.9% of the ICL group patients and 96.9% of the SMILE group patients were satisfied with the visual outcome. Further, 96.9% and 93.8% of the ICL group and SMILE group patients, respectively, would recommend the procedure to others with similar conditions.

## Discussion

With advances in corrective refractive technology, postoperative visual quality has become a top priority for research. In this study, both objective and subjective visual parameters were assessed including the safety, efficacy, HOAs, the OSI, and patient-reported questionnaire in an effort to give a comprehensive comparison.

In this study, the safety index was 1.22 ± 0.21 in the ICL group and 1.15 ± 0.14 in the SMILE group with no intraoperative or postoperative complications. No eyes from either group lost two or more lines of CDVA over 4 years, demonstrating the safety of both procedures. In addition, the efficacy index was comparable between the SMILE and ICL groups. Yan et al.[20] reported the two-year safety and efficacy indexes as 1.24 ± 0.26 and 1.03 ± 0.23, respectively, after EVO-ICL implantation. Yang et al.[21] reported that the safety index was 1.23 ± 0.22, and the efficacy index was 1.04 ± 0.16 4-year postoperatively. Blum et al. reported SMILE [2] provides good long-term safety and efficacy of refraction in a 10-year observation. The meta-analysis conducted by Chen et al. revealed that ICL V4c implantation demonstrated a significantly higher safety index than SMILE [22].

SMILE exhibited a slightly superior predictability profile in this study. 81% and 72% of eyes were within ± 0.50 D of the attempted spherical equivalent correction after SMILE and EVO-ICL implantation, respectively (P < 0.01). The postoperative mean residual cylinder after SMILE was slightly better than after EVO-ICL implantation. This is partly because in the ICL group, eyes with non-TICL might increase the mean residual SE owing to the uncorrected cylinder refraction. Despite the slight differences between the two procedures in terms of predictability, this study results still had a good performance comparable with previous study findings. In long-term studies of ICL implantation, Yang et al. [21] observed that 79% eyes had within ± 0.50 D of the attempted correction at 4 years. Igarashi et al.[7] observed 82.9% at 4 years and 68.3% at 8 years, and Alfonso et al.[6] observed 38.0% at 5 years. After SMILE, Han et al.[23] reported 80% eyes within ± 0.50 D of the attempted correction at 3 years, and Li et al.[24] reported 90% at 5 years. Our results were consistent with those of previous studies and demonstrated the safety and effectiveness of the two techniques.

There are concerns about the long-term endothelial cell loss postoperatively. In an FDA clinical study, Sanders et al.[25] found a cumulative endothelial cell loss of between 8.4% and 8.9% over the first 3 years after ICL implantation. Igarashi et al.[7] reported 6.2% of endothelial cell loss 8 years after surgery. Particularly focused on EVO-ICL, Lisa et al.[26] reported a 1.7% reduction in ECD at 1 year. Yang et al.[21] reported a 4.03% reduction at 4 years and demonstrated that the anterior segment biometric parameters, especially the vault, induced a decline in ECD. Our results showed a similar degree of endothelial cell loss (5.6% ± 10.7%) with the previous studies.

In this study, the postoperative ocular HOAs and OSI were assessed for the objective optical performance after the two procedures. Eyes treated with SMILE showed significantly higher amount of spherical aberrations and coma than those treated with ICL. ICL implantation for high myopia has shown excellent aberrometric control compared with other cornea refractive surgeries such as FS-LASIK or PRK [27, 28]. It also showed superiority to SMILE. Siedlecki et al.[14] reported that SMILE-treated eyes showed significantly higher degree of spherical aberration, coma, and total HOAs than ICL-treated eyes at 1 year. Our previous study based on myopic patients has documented that SMILE-treated eyes induced significantly higher amounts of coma (both vertical coma and horizontal coma) than ICL-treated eyes [29]. Few surgically induced HOAs after ICL implantation may be attributed to the retention of unchanged corneal biomechanics and a negative spherical aberration of ICLs [12]. In contrast, SMILE may change the natural asphericity of the cornea into a relatively oblate surface, especially for high-myopic eyes, resulting in more induced HOAs [12].

There were no differences in the OSI and other parameters between the groups. OSI in both groups (1.3 ± 0.5 in the SMILE group and 1.3 ± 0.7 in the ICL group) was comparable with that reported in previous studies. For ICL-treated eyes, the short-term OSI was 1.08 in Huseynova’s study [18] and was 1.16 in Kamiya’s study [30]. Our previous study found that the mean OSI was 0.71 ± 0.38 at 18 months after SMILE with lower results than that reported in the present study. An OSI > 1.5 indicates significant light scattering [31]. The mean postoperative value for each group was not over 1.5, which suggested satisfying retinal image quality. Regarding the MTF_cutoff_ value, Kamiya et al.[30] and Qin et al.[13] reported 26.21 cpd and 48.96 cpd, respectively, which were lower and higher, respectively, than that reported in the current study (30.46 cpd). In SMILE, our group found that the MTFcutoff was 38.20 cpd 12-month postoperatively and 37.81 cpd 18-month postoperatively,[32] which were higher that that reported in this study (27.95 cpd). The difference may be due to the longer follow-up period, which needs to be discussed further.

Halo was the most commonly reported night vision disturbance in the ICL group (91%), although it was considered to be non-distressing in more than 90% of cases in both groups. Halos after conventional ICL implantation have been reported in previous studies [10, 11, 33] and were found to be associated with differences between the mesopic pupil size and ICL optic zone diameter, and white-to-white diameter of the cornea [11]. Regarding EVO-ICL, apart from the edge of the optical zone, the cylindrical inner wall of the central hole can be an additional optical surface causing light phenomena [34, 35]. Lin et al. [36] proposed that ICL implantation led to increased peripheral distortion, which may have an impact on postoperative visual quality in patients. Siedlecki et al.[14] reported a prevalence of 80% for halos at 2 years after EVO-ICL implantation, and Wei et al.[12] reported it as 93.5% at 3 months, which were consistent with our findings.

Previous studies found only very weak and probably clinically irrelevant associations between HOAs and subjectively perceived visual quality after SMILE [12, 37]. This finding may explain the same high-quality subjective visual quality obtained even though the HOA was higher than ICL after SMILE.

This study has some limitations. First, the sample size of both groups was relatively small owing to the long-term follow-up period. In order to overcome this limitation and maximize the available data, we included both eyes of the patients in the study and employed the Generalized Estimating Equations (GEE) framework for data analysis. By utilizing the GEE equations, we aimed to minimize the statistical errors associated with the inclusion of binocular data. Second, only eyes with SE ranging from − 10.00 D to -6.00 D were included owing to the restriction in the application of SMILE. These findings cannot be extrapolated to patients with SE over − 10.00 D. Third, the lack of preoperative data in OQAS and HOAs analysis limited us from exploring the change between pre- and postoperative optical system and finding the associations between the changes and the visual complaints. A strength of the study was its comprehensive report, in both objective and subjective manner, of the four-year long-term visual outcomes after SMILE and ICL implantation in correcting high myopia. This study may help surgeons make clinical decisions for their patients with high myopia.

## Conclusions

Both SMILE and EVO-ICL implantation demonstrated safety and efficacy in correcting high myopia. SMILE showed slightly better long-term predictability. Mild postoperative visual disturbances were observed after ICL and SMILE at 4 years, which indicated that although the visual disturbances were not severe, the long-term duration and high prevalence were significant.

## Electronic supplementary material

Below is the link to the electronic supplementary material.

Supplementary Material 1

## Data Availability

All data generated or analyzed during this study are included in this article.
