# Supplementary material for: Four-year visual outcomes and optical quality of SMILE and implantable collamer lens V4c (EVO-ICL) implantation for high myopia: a retrospective study
Source: BMC Ophthalmol. 2023 Jul 31;23:341. doi: 10.1186/s12886-023-03050-9 (PMC10392000; doi:10.1186/s12886-023-03050-9)
Supplement: Supplementary file 1 — Supplementary Material 1 [file 12886_2023_3050_MOESM1_ESM.docx]

**Supplementary Table 1** Patient questionnaire

| **Parameter** |
| --- |
| 1 Do you experience any night vision disturbance currently? If **YES**, which eye?  □ No □ Yes (operated eye, unoperated eye, or both eyes) |
| 2 During the last week, have you experienced interocular visual differences at night? If **YES**, which eye is better in term of night vision?  □ No □ Yes (operated eye or unoperated eye) |
| 3 During the last week, have you experienced glare at night? If so, which eye? And rate it as mild, moderate, or severe.  □ No □ Yes (operated eye, unoperated eye, or both eyes) □ Mild □ Moderate □ Severe |
| 4 During the last week, have you experienced halos (rings around lights) at night? If so, which eye? And rate it as mild, moderate, or severe.  □ No □ Yes (operated eye, unoperated eye, or both eyes) □ Mild □ Moderate □ Severe |
| 5 During the last week, have you experienced starburst around lights at night? If so, which eye? And rate it as mild, moderate, or severe.  □ No □ Yes (operated eye, unoperated eye, or both eyes) □ Mild □ Moderate □ Severe |
| 6 During the last week, have you experienced any visual distortion as you normally function at night? If so, which eye? And rate it as mild, moderate, or severe.  □ No □ Yes (operated eye, unoperated eye, or both eyes) □ Mild □ Moderate □ Severe |
| 7 The overall satisfaction score (on a scale of 1–10, 1 = low satisfaction and 10 = high) with your refractive surgery outcomes is ___. |

Mild: symptoms noted to affect light sources, but functions were not interfered; Moderate: symptoms noted and usual activity were affected, especially when driving or looking at light sources; Severe: certain activities, such as driving or looking at light sources, were restrained by symptoms
